# First report of canine Chagas disease on the Caribbean Island of Trinidad

**DOI:** 10.1590/0074-02760250199

**Published:** 2026-01-30

**Authors:** Rod Suepaul, Azad Mohammed, Nicole L Gottdenker, Indira Pargass, Christopher Oura, Adesh Ramsubhag, Lana Gyan, Vrijesh Tripathi, Jennifer K Peterson

**Affiliations:** 1 University of the West Indies, School of Veterinary Medicine, St. Augustine, Trinidad and Tobago; 2 University of the West Indies, Department of Life Sciences, St. Augustine, Trinidad and Tobago; 3 University of Georgia, College of Veterinary Medicine, Department of Veterinary Pathology, Athens, GA, USA; 4 Ministry of Agriculture Lands and Fisheries, Animal Production and Health Division, Champs Fleurs, Trinidad and Tobago; 5 University of the West Indies, Department of Mathematics and Statistics, Trinidad and Tobago; 6 University of Delaware, College of Agriculture and Natural Resources, Department of Entomology & Wildlife Ecology, Newark, DE, USA

**Keywords:** canine Chagas disease, Trypanosoma cruzi, triatomine bugs, Trinidad and Tobago

## Abstract

**BACKGROUND:**

Chagas disease (CD) is a vector-borne infection caused by *Trypanosoma cruzi*, a kinetoplastid parasite of mammals. *T. cruzi* is transmitted by triatomine bugs throughout the Americas and some Caribbean islands. On the Caribbean island of Trinidad, *T. cruzi* has been isolated from triatomine bugs in several residential areas where dogs are a common pet. However, canine *T. cruzi* infection in Trinidad has never been studied.

**OBJECTIVES:**

We aimed to demonstrate that canine CD does occur in Trinidad through a review of veterinary records from the years 2008-2023.

**METHODS:**

We reviewed 3,923 case reports from Trinidad veterinary clinics for canine Chagas cases diagnosed through histological evaluation, necropsy, blood smear evaluation, and/or polymerase chain reactions (PCR).

**FINDINGS:**

We identified 13 confirmed and two suspected canine CD cases. Animal ages ranged from five weeks to 14 years old, with four (27%) being less than one year old, including the pup of a *T. cruzi*-infected dam. Breed varied, although one-third (5/15) were hounds. Clinical signs ranged from asymptomatic (43%; 6/14) to severely ill with limb paresis (21%; 3/14). Seven of the fifteen (47%) dogs died, and three more (20%) were euthanized. Myocarditis with visible amastigote forms were found in two-thirds (9/15) of dogs.

**MAIN CONCLUSIONS:**

Our findings highlight a need for increased awareness of CD among dog owners and veterinarians in Trinidad.

*Trypanosoma cruzi* is a vector-borne parasite of mammals, and the causative agent of Chagas disease (CD) in humans and dogs.^1^ ^,^ ^2^ ^,^ ^3^ The arthropod vectors of *T. cruzi* are hematophagous insects called triatomine bugs that transmit the parasite stercorally (*i.e.*, in their excrement and not in their saliva). Insectivorous animals, including dogs, can also acquire *T. cruzi* orally through the consumption of *T. cruzi*-infected triatomine bugs, which is more efficient than stercoral transmission and can cause severe, acute disease.^4^ ^,^ ^5^ ^,^ ^6^ ^,^ ^7^ Other modes of *T. cruzi* transmission in dogs include transplacental transmission from mother to fetus.

Vector-borne *T. cruzi* transmission is broadly classified into domestic and sylvatic cycles. Domestic *T. cruzi* transmission occurs near or inside human dwellings, with the parasite circulating through triatomine bug vectors, domestic and synanthropic mammals, and humans.^8^ Sylvatic *T. cruzi* transmission occurs among mammals living in wild ecotopes and does not involve humans.^9^ There are also peri-domestic cycles, which involve some degree of overlap between domestic and sylvatic cycles, and involve wild mammalian species that thrive in human-dominated environments, such as raccoons and opossums.^10^ ^,^ ^11^ ^,^ ^12^ Domestic dogs are considered sentinels for human CD in domestic environments, due to their close relationship with humans and their propensity to eat insects, including triatomine bugs.^13^ ^,^ ^14^ ^,^ ^15^ ^,^ ^16^ ^,^ ^17^ Dogs that sleep outdoors and accompany their owners when hunting wild animals are especially vulnerable to *T. cruzi* infection.^17^ Kennels or shelters housing multiple dogs in regions with triatomine bugs also pose an increased risk of canine *T. cruzi* infection.^14^ ^,^ ^18^ ^,^ ^19^ ^,^ ^20^ ^,^ ^21^

The progression of canine CD follows a similar pattern to the disease in humans, comprising acute, indeterminate, and chronic phases.^1^ ^,^ ^3^ ^,^ ^22^ ^,^ ^23^ The acute phase occurs shortly following infection. Clinical signs of the acute phase include diarrhea, lethargy, swollen lymph nodes, fluid retention, ascites, and in severe cases, acute myocarditis and sudden death.^4^ ^,^ ^24^ ^,^ ^25^ Dogs that recover from acute infection move into an asymptomatic indeterminate phase with a subpatent parasitemia.^26^ The duration of the indeterminate phase is variable; some dogs will remain in this phase indefinitely, while others will progress to the chronic phase within an estimated eight to 36 months.^22^ Clinical signs of the chronic phase include cardiac arrhythmias and signs of cardiac compromise or heart failure, including lethargy, fainting, fluid buildup in the abdomen or lungs. Clinical signs in dogs can vary with age, with young puppies exhibiting more severe symptoms. Sudden death can occur in both the acute and chronic phases.^1^

Pathologic changes in dogs with CD include myocarditis, hepatomegaly, ascites, and cardiac enlargement and dilatation.^1^ ^,^ ^22^ ^,^ ^24^ ^,^ ^27^ Megaoesophagus and megacolon may also be present in both acute and chronic phases.^28^ Histologic changes include intracytoplasmic amastigote pseudocysts accompanied by lymphoplasmacytic to histiocytic inflammation, cardiomyocyte necrosis, and replacement fibrosis.^1^ ^,^ ^25^ ^,^ ^26^ ^,^ ^27^ ^,^ ^29^ ^,^ ^30^ *T. cruzi-*infected hearts without detectible amastigotes may still yield positive results in polymerase chain reactions (PCRs) targeting *T. cruzi* DNA, as the parasite is not always readily detectable upon histologic examination of chronically infected dogs.^23^ ^,^ ^26^ ^,^ ^31^

There is currently no canine vaccine against CD, although development efforts are underway.^23^ ^,^ ^32^ Antiparasitic medication for canine CD has been explored with mixed results, and treatment of the disease generally consists of symptom management; there is no cure for chronic canine CD.^23^ ^,^ ^31^ ^,^ ^33^ Prognosis and survival of *T. cruzi*-infected dogs varies and not all infected dogs develop disease.^23^ ^,^ ^25^ ^,^ ^34^ ^,^ ^35^

*Chagas disease in Trinidad* - Although well-studied in South and Central America, CD is overlooked in the Caribbean Islands, despite evidence of triatomine bugs and *T. cruzi* in several of the islands.^36^ ^,^ ^37^ ^,^ ^38^ ^,^ ^39^ ^,^ ^40^ ^,^ ^41^ On the island of Trinidad in the dual island nation of Trinidad and Tobago, there are six described triatomine bug species. The most common triatomine species is *Panstrongylus geniculatus* (Fig. 1; reviewed in Suepaul 2025).^41^ A recent survey of *P. geniculatus* found in and around households in Trinidad found *T. cruzi* infection in over 80% of specimens tested.^38^ Recent detection of two triatomine bug species in Tobago indicates that *T. cruzi* likely circulates there as well, although studies are needed to confirm its presence.^41^

**Fig. 1:**
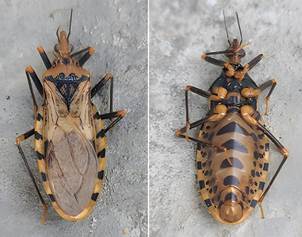
*Panstrongylus geniculatus*, the main *Trypanosoma cruzi* vector in Trinidad and Tobago. Photo taken by Dr Amy Deacon and originally published in Suepaul et al.^42^

In Trinidad, as in many regions, dogs are commonly an integral member of human households, serving purposes that include companionship, hunting, and protection. Dogs from households in Trinidad that are located near triatomine bug nidi and dogs that are used for hunting wildlife reservoirs of *T. cruzi,* including nine-banded armadillos (*Dasypus novemcinctus*) and opossums (*Didelphis marsupialis*) could be at an increased risk of acquiring *T. cruzi* infection.^42^ ^,^ ^43^ However, local canine *T. cruzi* infection remains unexamined. Therefore, we investigated canine CD in the island of Trinidad using 3,923 historical case records from two veterinary diagnostic clinics. Our objectives were to (i) demonstrate that *T. cruzi* transmission occurs in dogs in Trinidad; (ii) document the disease and its pathology in locally transmitted cases; (iii) raise awareness of canine CD in TT to facilitate the detection of suspected cases; (iv) provide baseline data to guide future research of canine *T. cruzi* infections in Trinidad and Tobago.

## MATERIALS AND METHODS

*Historical records search* - We conducted a retrospective study of canine necropsy and blood submission records from the University of the West Indies School of Veterinary Medicine Pathology Laboratory, as well as necropsy records from the Trinidad and Tobago Ministry of Agriculture Veterinary Diagnostic Laboratory (VDL). We searched all available digital diagnostic records (N = 3,023) of dogs submitted to the VDL between January 2008 and December 2023. Canine necropsy records were examined for myocarditis and other lesions or diagnostic results consistent with *T. cruzi* infection. Clinical records were also screened for cases where the submitting veterinarian had tested blood for *T. cruzi*, which is not routinely performed otherwise. All cases were likely locally acquired, as no history of travel outside the country was reported.

*Necropsy procedures* - Necropsy procedures reported in each record consisted of routine gross examination of all internal organs except for the central nervous system, which was only examined when indicated by the clinical history. Samples of any lesions detected, as well as representative sections of liver, lung, and kidneys were placed in 10% buffered formalin. After a 48-hour fixation, the tissues were trimmed and embedded in paraffin blocks. Sections 4 µm thick were placed in glass slides, deparaffinized, and routinely stained with hematoxylin and eosin for microscopic examination.

*Polymerase chain reaction detection methods* - In cases where PCR was used to detect *T. cruzi* infection, the assay consisted of DNA extracted from each sample (blood or representative organ) amplified in a PCR using the TcZF/R primers, which amplify a 182 bp region of *T. cruzi* nuclear DNA (5’-GCTCTTGCCCACAAGGGTGC-3’ and 5’-CCAAGCAGCGGATAGTTCAGG-3’).^44^ ^,^ ^45^

## RESULTS

*Overview* - Out of the 3,923 canine records reviewed, we found two suspected and 13 confirmed cases of canine CD. Dog age ranged from five weeks to 14 years old, with four (27%) being less than one year old, including the five-week-old pup of a *T. cruzi*-infected dam. Dog breed varied, although one-third (5/15) were hounds. Clinical signs ranged from asymptomatic (43%; 6/14) to severely ill with limb paresis (14%; 2/14). Seven of the fifteen (47%) dogs died and three more (20%) were euthanized. Myocarditis with visible amastigote forms were found in two-thirds of dogs (9/15).

*Case descriptions* - The following is a summary of the cases identified, presented in in chronological order. A complete and detailed list of case descriptions and corresponding pathology is available in Table.

**TABLE:** Descriptions for the 15 canine *Trypanosoma cruzi* infection cases identified in Trinidad

Case #	Year	Signalment	Origin	History	Gross	Histology	PCR +	Notes
1^*^	2008	4 year old, intact male, Dalmatian	Diego Martin	Sudden death	Moderate pulmonary oedema; pale heart	Severe necrotizing lymphoplasmacytic myocarditis with intracytoplasmic amastigotes	NA	
2^*^	2013	9 mo., intact female, Rottweiler	Maraval	Sudden death	No significant lesions	Severe necrotizing lymphoplasmacytic myocarditis with intracytoplasmic amastigotes	NA	
3	2018	5 wk., intact male, Cocker Spaniel	Diego Martin	On medication for tick fever (doxycycline) enrocillina and Alban© for hookworm for two days before it died.	Moderate ascites; hydropericardium	Myocarditis severe, lymphoplasmocytic and histiocytic, necrotising with intracytoplasmic amastigotes	Blood	
4^+^	2018	Adult, intact female, Cocker Spaniel	Diego Martin	Dam to above	NA	NA	Blood	
5	2019	1.5 yr old, intact male, foxhound	Cunaripo	Anorexia with marked weakness and hindlimb paresis. Euthanized.	*T. cruz*i forms visible in blood smear; pale heart muscle	Myocarditis with intracytoplasmic amastigotes. Moderate multifocal lymphoplasmocytic and monocytic lumbar myelitis	Spleen and heart	
6	2020	Adult, intact male, Hound	Not Stated	No Chagas symptoms; taken to clinic because it was hit by a car; luxation of T11 on spinal cord and comminuted fracture of femur. Euthanized.	Fractures and splenic nodular hyperplasia	No cardiac lesions observed	Spleen and heart	
7	2020	Adult, intact female, Hound	Not Stated	Owner submitted carcass for necropsy. No history.	Multifocal moderate renal fibrosis and mild pulmonary oedema.	No cardiac lesions observed	Spleen and heart	
8	2020	4 mo., intact female, Hound	Cunaripo	Submitted for necropsy. Pup was not eating and had pale mm. Swollen abdomen.	Haemorrhagic enteritis, hydrothorax and ascites, subcutaneous oedema. GIN eggs 3+ -severe endoparasitism	No cardiac lesions observed.	Spleen and heart	Lived near positive case from 2019.
9	2020	3 mo., intact male, Husky	South Oropouche	Received 3rd round of routine vaccinations^^^ on 20/06/2020. Refused meal the next day. Started eating and drinking little. Was placed on oral rehydration, but continued to not eat or drink and died the same month (June).	Severe anaemia, mild hydrothorax and ascites	Myocarditis with myocardial degeneration and necrosis. Severe, acute, diffuse, with intra-myocytic protozoal amastigotes.	Heart and blood	
10	2020	Adult, intact male, Hound	Mamoral	Developed respiratory distress and died.	Pulmonary congestion and oedema, moderate and diffuse	Moderate multifocal lymphoplasmacytic and histiocytic fibrotic myocarditis.	Heart	From an armadillo hunting pack.
11	2021	Adult (2 yr), Spayed female, mixed breed	Coal Mine	Paresis in left forelimb for approximately 2 mo., progressing to quadriparesis. Unable to walk; unresponsive to corticosteroid, NSAID and B vitamin therapy. Euthanasized	NA	NA	Blood	From household where > 80 triatomine bugs captured since 2016
12^+^	2023	Adult (14 yr), intact male, German shepherd	Manzanilla	Hindlimb paresis, urinary tract infection and prostatitis for several weeks.	NA	NA	Blood	Owner found triatomine bugs feeding on dog.
13^+^	2023	Adult (7 yr), Spayed female, mixed breed	Manzanilla	No clinical disease	NA	NA	Blood	From same yard as above
14^+^	2023	Adult (2 yr), Spayed female, Malinois	Manzanilla	No clinical disease	NA	NA	Blood	From same yard as above
15^+^	2023	Adult (5 yr), Spayed female, mixed breed	Manzanilla	No clinical disease	NA	NA	Blood	From same yard as above

NA: indicates that procedure was not carried out; *Suspected cases (No polymerase chain reaction - PCR - confirmation); ^+^Still alive at time of case record; ^^^Routine vaccinations in Trinidad consist of vaccines against the following: Canine distemper virus, canine adenovirus type 2, canine parainfluenza virus, canine parvovirus, and *Leptospira* spp.

*2008-2018* - The first canine CD cases identified in this review occurred in 2008 and 2013 and involved a four-year-old male Dalmatian and a nine-month-old female Rottweiler, respectively. Both died suddenly. Histological examination revealed that both animals had a severe lymphoplasmacytic and histiocytic myocarditis with visible protozoal amastigotes within the cytoplasm of cardiac myocytes, leading the diagnosing veterinarians to suspect *T. cruzi* infection. These are the only cases where PCR-based diagnostics were not done to confirm the diagnosis.

**Fig. 2:**
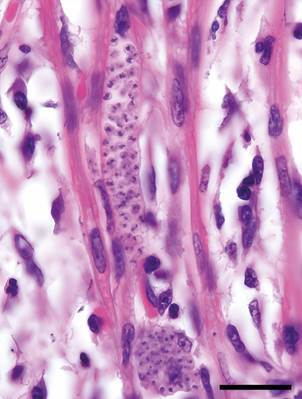
myocarditis with intra-cardiomyocyte pseudocyst containing amastigotes from five-week-old Cocker Spaniel that died in 2018 (Case 4). Polymerase chain reaction (PCR) was *Trypanosoma cruzi* positive. Hematoxylin and Eosinstain (H&E) stain, 100x magnification. Bar = 20µm.

The third and fourth cases occurred in 2018 when a five-week-old male Cocker Spaniel died two days after beginning treatment for both *Rickettsia* bacterial infection and hookworm. Necropsy revealed myocarditis and intracytoplasmic protozoal amastigotes in cardiac myocytes (Fig. 2), and blood from both the pup and its mother (who was not visibly sick) tested positive for *T. cruzi* via PCR.

**Fig. 3:**
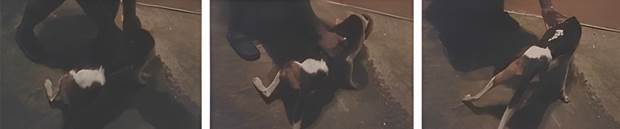
stills from a video of the foxhound with limb paresis (Case 5). Given the presence of *Trypanosoma cruzi* amastigotes in the heart, parasite forms observed in the blood, and polymerase chain reaction (PCR)-positivity of the heart and spleen, the cause of the hindlimb paresis in the animal was likely Chagasic myelitis. However, direct evidence of *T. cruzi* in the spinal cord was not collected; PCR was conducted just with the heart and the spleen tissues, and immunohistochemistry was not carried out [Supplementary data (video) ].

**Fig. 4:**
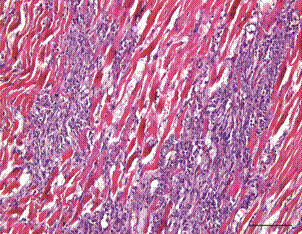
chronic myocarditis with fibrosis from adult hound diagnosed in 2020 (Case 10). Heart tissue was polymerase chain reaction (PCR) positive for *Trypanosoma cruzi* infection. Hematoxylin and Eosinstain (H&E) stain, 20x magnification. Bar = 100µm.

*2019-2023* - Diagnosed canine Chagas cases increased during this period, likely due to increased awareness of *T. cruzi* infection among veterinary clinic staff, as follows:

- The fifth case was from 2019, where an 18-month-old male foxhound was brought in with anorexia, marked weakness, and hindlimb paresis [Fig. 3 and Supplementary data (video)]. The owner decided to euthanize the dog. *T. cruzi* forms were observed in blood smears from the dog, indicative of acute and recent infection. Histological examination revealed myocarditis and intracytoplasmic amastigotes; DNA from heart and spleen tissues were PCR-positive for *T. cruzi*. Given the presence of *T. cruzi* amastigotes in the heart and PCR-positivity of the heart and spleen, it is highly likely that the myelitis detected was a result of *T. cruzi* infection and the cause of the hindlimb paresis in the animal. However, direct evidence of *T. cruzi* in the spinal cord was not collected, as PCR was done just on the heart and the spleen tissues and immunohistochemistry analyses were not carried out.

- The following year (2020), three adults and two pups under six months old were diagnosed with canine CD (cases 6-10, Table). Two of the dogs were hunting dogs, one of which was from a pack specializing in armadillo hunting. Armadillos are a *T. cruzi* reservoir species and a common host for *P. geniculatus*, the most common triatomine bug species in Trinidad.^46^ ^,^ ^47^ The dog, which died suddenly after displaying respiratory distress, had pathological signs of histiocytic fibrotic myocarditis suggestive of chronic canine CD (Fig. 4). All five dogs diagnosed in 2020 were PCR-positive for *T. cruzi* with DNA extracted from heart tissue. Three of these dogs were also positive with DNA extracted from spleen tissue. One pup, a four-month-old hound, was from a home located near the 18-month-old foxhound diagnosed with canine CD in 2019.

- A single canine Chagas diagnosis (case 11, Table) was made in 2021. The animal was a two-year-old mixed breed female that presented with paresis in her left forelimb for approximately two months preceding and progressing to quadriparesis. The animal was unable to walk and unresponsive to corticosteroid, NSAID and B vitamin therapy; the owner eventually decided to euthanize her. The dog resided in a household where over 80 triatomine bugs have been captured since 2016.

- The final four dogs were diagnosed with *T. cruzi* infection in 2023, and they were all from a single household. After viewing an educational flyer on CD distributed by our group to veterinary clinics throughout Trinidad, the dog owner discovered *T. cruzi* vector species *P. geniculatus* feeding on his sick 14-year-old German Shepard. The dog tested positive for *T. cruzi* infection and the owner subsequently requested that his four other dogs (all asymptomatic) be tested as well*.* Three of these four dogs were PCR-positive for *T. cruzi* infection, meaning that four of the five dogs from this household were infected with *T. cruzi*, although only one was symptomatic.

## DISCUSSION

*Trypanosoma cruzi* has been detected in Trinidad since 1958, but the parasite has not been studied in dogs on the island until now.^41^ Here, we provide the first reports of locally transmitted canine *T. cruzi* infections. Severity between cases varied, but still conformed to the profile of canine CD. Three of the most severe cases occurred in pups under nine months old, which aligns with previous observations of younger dogs being more likely to show signs of acute disease.^22^ Our findings suggest the existence of an active *T. cruzi* transmission cycle in Trinidad involving canines.

*Variation in clinical signs reflect challenges in canine Chagas surveillance and diagnosis* - Three of our youngest cases had non-specific symptoms that were initially mistaken for a different, more frequently observed condition, which is a common challenge in identifying canine CD.^48^ Case 3, the five-week-old Cocker Spaniel pup, was initially thought to have tick fever and hookworm. Case 9, the three-month-old Husky pup that would not eat or drink, which was assumed to be a side effect of recent vaccinations. Unfortunately, both animals died within days of illness onset and necropsy examination revealed that both had had severe protozoal myocarditis. In addition, the symptoms of *T. cruzi* infection can be masked by a concomitant infection, which we observed in the four-month-old hound pup (case 8). The animal was severely infected with gastrointestinal helminths, which possibly masked her *T. cruzi* infection, which has been observed in other studies of *T. cruzi*-helminth coinfections.^49^ ^,^ ^50^

Another challenge with CD surveillance in both humans and dogs is that the chronic form of the disease often goes undiagnosed due to undetectable parasitemia or subclinical infection.^1^ ^,^ ^26^ ^,^ ^27^ In our study, we observed several subclinical cases that would have gone undetected had it not been for other events prompting examination of the animals. For example, in the 2020 case of the hound from Mamoral (case 10), the animal had no observable *T. cruzi* forms even though the degree of fibrosis indicated that the changes were chronic and PCR detected *T. cruzi* DNA. A second hound from 2020 (case 6) was taken to the clinic because it was hit by a car.

Finally, although CD presents with a wide range of symptoms, most studies of *T. cruzi* infection in dogs focus on cardiac manifestations, which may result in misdiagnoses in dogs displaying gastrointestinal (GI) or neurological alterations, which are reported in a small number of canine Chagas cases.^3^ ^,^ ^29^ ^,^ ^51^ We found one dog with neurological signs (case 5, a foxhound with multifocal myelitis in 2019) although it was accompanied by classic signs of acute CD- circulating trypomastigotes, cardiac lesions, and positive PCR of the spleen and heart. No GI examinations were reported in the records we reviewed.

*Trypanosoma cruzi- infected mother and dam present possibility of transplacental T. cruzi transmission* - Our data included a *T. cruzi-* infected mother and pup (cases 4 and 3, respectively), presenting the possibility that the pup was infected with *T. cruzi* transplacentally. Vectorial transmission is also possible- the pup died at five weeks old, which is enough time for the pup to have acquired *T. cruzi* vectorially and developed peak parasitemia (~17 days post-infection).^22^ As it stands, we have no direct evidence that clearly points to either transmission route and evidence of transplacental transmission is circumstantial. There are few studies of transplacental *T. cruzi* transmission in dogs, but one recent study of fetuses from naturally infected dams found a *T. cruzi* transmission frequency of 59%.^52^ This transmission rate is much higher than that observed in humans, which has been estimated to be around 6%.^53^ ^,^ ^54^ This is a notable difference given that the disease progression and pathology of CD in dogs and humans is otherwise considered to be quite similar.^1^ ^,^ ^3^ ^,^ ^23^

*Environmental factors* - Our findings suggest that in cases where nonspecific or unusual clinical pathology is observed, environmental factors of the dog’s home and activities (*e.g.*, hunting, other outdoor activities, etc.) should be considered. The two hounds with histological lesions (cases 5 and 10, Table) were housed outdoors in kennels with multiple dogs kept close together, which are risk factors for canine CD.^19^ ^,^ ^20^ ^,^ ^55^ ^,^ ^56^ The main prey of Case 10, a hunting hound from Mamoral, was armadillo, which is a *T. cruzi* reservoir that often cohabitates with triatomine bugs in its burrows.

The presence of triatomine bugs at a dog’s home or residence are also important to consider, as highlighted by the mixed breed female from 2021 (case 11), which resided in a location where over 80 triatomine bugs have been collected since 2016. This animal presented with severe, nonspecific disease that did not conform to the classic Chagas pathology, yet whole blood from the dog tested positive for *T. cruzi*. In addition, triatomine bugs were found biting the dog from case 12 at its residence, which it shared with the dogs from cases 13-15. Taken together, these cases suggest active vector-borne *T. cruzi* transmission to canines occurring at their places of residence. Interestingly, the dog owner from cases 12-15 shared that he was bitten by a triatomine bug vector in the past and was unable to find any information about what had bit him. This anecdote highlights the need for publicly available information on both canine and human CD in Trinidad.^41^

*In conclusion* - Here, we provide evidence of locally acquired canine CD in Trinidad. As mentioned, dogs are considered a sentinel species for *T. cruzi* transmission to humans in some regions due to their close contact with people and propensity to consume insects.^10^ ^,^ ^17^ ^,^ ^57^ Keeping in mind that canine *T. cruzi* infection does not extrapolate directly to human CD risk, our findings merit further research into this question as it plays out within the context of *T. cruzi* transmission in Trinidad. Moreover, our results highlight the need for increased CD surveillance and research in Trinidad and Tobago in order to estimate both human and veterinary risk and develop appropriate prevention and control strategies.

## Supplementary Materials

https://youtube.com/shorts/-qP7R9fH2bU

## Data Availability

The contents underlying the research text are included in the manuscript.
